# Tissue-resident memory T cells in chronic liver diseases: Phenotype, development and function

**DOI:** 10.3389/fimmu.2022.967055

**Published:** 2022-09-12

**Authors:** Yikang Li, Zhengrui You, Ruqi Tang, Xiong Ma

**Affiliations:** Division of Gastroenterology and Hepatology, Key Laboratory of Gastroenterology and Hepatology, Ministry of Health, State Key Laboratory for Oncogenes and Related Genes, Renji Hospital, School of Medicine, Shanghai Jiao Tong University, Shanghai Institute of Digestive Disease, Shanghai, China

**Keywords:** tissue-resident memory T cells, liver, chronic hepatitis B virus infection, malaria, autoimmune hepatitis, nonalcoholic fatty liver disease, hepatocellular carcinoma

## Abstract

Tissue-resident memory (T_RM_) T cells are a unique subset of memory T cells that are critical for the first line of defense against pathogens or antigens in peripheral non-lymphoid tissues such as liver, gut, and skin. Generally, T_RM_ cells are well adapted to the local environment in a tissue-specific manner and typically do not circulate but persist in tissues, distinguishing them from other memory T cell lineages. There is strong evidence that liver T_RM_ cells provide a robust adaptive immune response to potential threats. Indeed, the potent effector function of hepatic T_RM_ cells makes it essential for chronic liver diseases, including viral and parasite infection, autoimmune liver diseases (AILD), nonalcoholic fatty liver disease (NAFLD), hepatocellular carcinoma (HCC) and liver transplantation. Manipulation of hepatic T_RM_ cells might provide novel promising strategies for precision immunotherapy of chronic liver diseases. Here, we provide insights into the phenotype of hepatic T_RM_ cells through surface markers, transcriptional profiles and effector functions, discuss the development of hepatic T_RM_ cells in terms of cellular origin and factors affecting their development, analyze the role of hepatic T_RM_ cells in chronic liver diseases, as well as share our perspectives on the current status of hepatic T_RM_ cell research.

## Introduction

T cells are essential for building an effective immune response against pathogens or antigens. Once the pathogen breaks through the barrier tissue and invades the body, antigen-presenting cells (APC) capture the foreign antigen and then migrate to the local draining lymph nodes to activate naive T cells. Primed naive T cells subsequently proliferate and differentiate into effector T cells that migrate into inflamed tissues to eliminate pathogens (1). Among these effector T cells, a minor fraction persists and develops into memory T cells precursors after the pathogens are cleared. According to their unique patrolling properties, proliferative potential, and effector function, these memory T cell precursors eventually develop into circulating memory T cells and tissue-resident memory T (T_RM_) cells (2, 3). Circulating memory T cells include central memory (T_CM_) cells that target and patrol in the lymph node and egress to the blood after infection, and effector memory (T_EM_) cells that survey nonlymphoid peripheral tissues and enter the peripheral circulation thorough the lymphatic system (4). By contrast, T_RM_ cells almost not recirculate and are retained within tissues under homeostatic conditions (5).

Both CD8^+^ and CD4^+^ subpopulations of T_RM_ cells are detected at different tissue sites (6–8). CD8^+^ T_RM_ cells are well defined and enhance immune responses in peripheral tissues. However, the characteristics and functions of CD4^+^ T_RM_ cells remain largely unclear (9, 10). In general, T_RM_ cells primarily develop and persist in organs that are frequently exposed to pathogens or antigens, such as the liver, gut, skin and lung (11, 12). Among these organs, the liver is considered as a vital immune organ, and it is exposed to various pathogens and food antigens, that enter or re-enter the body *via* portal vein from the gastrointestinal tract and the systemic blood circulation.

Liver contains a large number of innate immune cells, including natural killer (NK) cells, NKT cells, γδ T cells, mucosal-associated invariant T cells, Kupffer cells, and dendritic cells (13). Interestingly, liver also include a number of liver-specific antigen-presenting cells, such as hepatic sinusoidal endothelial cells and hepatic stellate cells, which contribute to immune tolerance in the liver (13). Moreover, the hepatic specific immune microenvironment constructed by these immune cells promotes the generation of antigen-experienced T cells and T_RM_ cells involved in pathogen clearance or autoimmune responses against self-antigens (14). Importantly, liver T_RM_ cells perform an essential role in the first line of adaptive cellular defense while exposing to the cognate antigens in the liver (15, 16). Accordingly, the liver acts as an essential gatekeeper to prevent systemic infection and inflammation, while the liver T_RM_ cells contribute to the efficient eradication of pathogens as well as immune responses.

In this review, we primarily focus on phenotype and development of hepatic T_RM_ cells, mainly CD8^+^ T_RM_ cells, with emphasis on their protective roles in viral and parasite infection, non-alcoholic fatty liver disease (NAFLD), hepatocellular carcinoma (HCC) and liver transplantation, as well as their pathogenic roles in autoimmune liver diseases (AILD) (**Table 1**).

**Table 1:** Phenotype and clinical significance of liver CD8+ TRM cells in chronic liver diseases.

Chronic liver diseases	Phenotype	Clinical significance	Reference
HBV	CD69^+^CD103^+^CXCR3^+^CXCR6^+^CD39^+^PD1^+^BLIMP1^hi^HOBIT^+/lo^T-bet^lo^EOMES^lo^IL2^+^IFN-γ^+^perforin^+^	Virus-specific liver T_RM_ cells control viral replication, and contribute to the functional cure for HBV patients. Liver T_RM_ cells persist in the liver and provide long-term viral control in HBV patients.	(17–22)
HCV	CD69^+^CD103^+/-^CXCR6^+^S1PR1^lo^KLF2^lo^granzyme B^+^	Liver T_RM_ cells have specific activating and cytolytic potential for viral eradication.	(23–27)
Malaria (Murine study)	CD69^+^CD49a^+^LFA-1^+^CD101^+^CXCR3^+^ CX3CR1^lo^KLRG1^lo^CD107a^+^T-bet^+^EOMES^lo^IFN-γ^+^TNF-α^+^granzyme B^+^	Liver T_RM_ cells can directly kill *Plasmodium*-infected cells, thereby mediating protective immune responses. T_RM_-based vaccination strategies could hold remarkable promise in the prevention and treatment of malaria.	(16, 28–35)
AIH	CD69^+^CD103^+^CD49a^+^CXCR3^+^CXCR6^+^PD1^+^BLIMP1^hi^T-bet^lo^IL2^+^IL17^+^IFN-γ^+^ granzyme B^+^	Antigen-specific liver T_RM_ cells infiltration may serve as a new biomarker of pediatric acute liver failure (PALF) due to AIH. Histological remission in AIH patients is accompanied by a reduction in liver CD8^+^ T_RM_ cells, and liver T_RM_ cells may be an important factor in relapse after steroid discontinuation.	(36–38)
NAFLD (Murine study)	CD69^+^CD103^-^CXCR3^+^CXCR6^+^LAG3^+^CTLA4^+^FasL^+^TOX^+^EOMES^+^	Liver CD8^+^ T_RM_ cells promote fibrosis resolution by inducing apoptosis of predisposed activated hepatic stellate cells (HSCs), and may perform a protective role in resolving liver fibrosis of NASH.	(39)
HCC	CD69^+^CD103^+^PD1^+^LAG3^+^TIM3^+^CTLA4^+^T-bet^lo^EOMES^+^	Enrichment of liver T_RM_ cells are associated with better prognosis in HCC patients.	(19, 40–42)

## Phenotype of liver T_RM_ cells

The general characteristics of T_RM_ cells include their strategic positioning in the tissues and effector functions. However, despite T_RM_ cells share some similar features, the phenotype, such as surface markers and transcriptional profiles, and the underlying mechanisms for their generation and retention are highly heterogeneous in different tissues.

### Surface markers

It is considered that the surface markers contribute to the identification and maintenance of hepatic T_RM_ cells. Similar to other tissue-specific T_RM_ cells, hepatic T_RM_ cells downregulate the expression of tissue egression markers, like soingosine-1-phosphate 1 (S1PR1), and the homing receptors such as CD62L and CCR7 (43, 44). Furthermore, hepatic T_RM_ cells usually express some adhesion molecule and chemokine receptors, including CD69 (44), CD103 (17, 45), CD49a (36), CXCR3 (17, 23) and CXCR6 (46, 47), which are involved in their localization and maintenance in the hepatic sinusoids and portal veins.

The lectin CD69 is constitutive expressed on the majority of liver T_RM_ subsets. Upon exposure to antigens or pro-inflammatory mediators, the expression of CD69 is strongly upregulated on activated CD8^+^ T cells within peripheral tissues as a result of the downregulation of Krüppel-like factor 2(KLF2) (44, 48, 49). Meanwhile, as an antagonist of S1P1, CD69 complexs with S1P1 on the cell surface and leads to its internalization and degradation (50). Besides, CD69 also contributes to the retention status of hepatic T_RM_ cells by downregulating sphingosine 1 phosphate receptor (S1PR1)-mediated tissue egress (44). Therefore, it is likely to that its primary role is to restrict the egress of T_RM_ cells from the liver to the blood and lymphatic vessels.

CD103 is an α-chain of the integrin αEβ7. It is upregulated in activated peripheral CD8^+^ T lymphocytes upon exposure to TGFβ (51). CD103 is a receptor for E-cadherin, an adherens junction protein interlocking epithelial cells (52). Interestingly, E-cadherin is widely expressed by hepatocytes and cholangiocytes (36, 53, 54).The interaction of E-cadherin and CD103 expressing on the liver-infiltrating lymphocytes may be involved in positioning, adhesion and retention of hepatic T_RM_ cells (36). Furthermore, CD103 may define two different functional subsets of T_RM_ cells in human liver. The CD69^+^CD103^+^ subpopulations are antigen-specific autoreactive cytotoxic T cells in human liver, exhibiting more potent effector function than CD69^+^CD103^-^ counterparts (45, 55, 56). Interestingly, there are differences between mouse and human liver T_RM_ cells regarding CD103 expression. Interestingly, it appears that another liver-specific homing marker, lymphocyte function associated antigen 1 (LFA-1), rather than CD103, may be responsible for the retention of hepatic T_RM_ cells in mice (16, 57).

CD49a, another adhesion molecule of T_RM_ cells, is the α1 component of the integrin α1β1. CD49a pairs with integrin β1 to form the heterodimer VLA-1 which bind to collagen IV. This interaction is believed to be critical for retention of the resident population at the epithelium (58). In general, CD49a is upregulated following T cell activation and can be found on circulating T cells (59). Expression of CD49a contributes to protect cells from undergoing apoptosis (60). Importantly, blockade of CD49a with antibodies as well as genetic deletion of CD49a results in a diminution of T_RM_ cells (59, 61). However, CD49a was not essential for the recruitment of CD8 T cells to the lung in mice, but for their persistence as memory cells (59). Therefore, CD49a may promote the survival, retention or proliferation of T_RM_ cells. Moreover, CD49a may define different functional subsets of T_RM_ cells. In the skin, CD49a expressing CD8^+^ T_RM_ cells produce large amounts of IFN-γ, perforin and granzyme B, while CD49a negative counterparts prefer to produce IL17 (62). However, the effector function bias based on CD49a expression of liver T_RM_ cells have not been comprehensively interrogated.

Chemokines and chemokine receptors have been extensively used to describe the correct localization, residence and effector function of immune cells within lymphoid organs and non-lymphoid tissues (63). Despite their expressions on T_RM_ cells of different tissues have great heterogeneity, it is reported that the maintenance and effector function of T_RM_ require constant chemokine stimulation (64–66). Chemokine receptors CXCR3 and CXCR6 have been extensively reported to be constitutively expressed on the surface of intrahepatic T_RM_ cells (16, 17, 23, 46, 67). CXCR3 is a vital homing marker that may contributes to the retention of liver CD8^+^ T_RM_ cells. It binds to multiple chemokines, such as CXCL9, CXCL10 and CXCL11, which are predominantly secreted by monocytes, liver sinusoidal endothelial cells and fibroblasts (17). On the other hand, CXCR6 also plays an important role in the maintenance of liver T_RM_ cells (46, 68). CD8^+^ T cells lacking CXCR6 migrate to the liver normally after immunization, whereas perform a marked decrease capacity to form hepatic CD8^+^ T_RM_ cells and severely impairs their effector functions against infection in the liver (46). In addition, CXCR6 also contributes to the maintenance of liver T_RM_ cells *via* binding to CXCL16 secreted by liver sinusoidal endothelial cells (46, 68). These studies suggest that CXCR6 is essential for retention rather than recruitment of CD8^+^T cells to the liver. Additionally, deficiency of CXCR6 results in decreased survival of hepatic NKT cells patrolling the liver sinusoids, affecting hepatic intravascular immune surveillance (68).

### Transcriptional profiles

Besides surface markers, multiple transcription factors are involved in the regulation of the distinct features of liver T_RM_ cells.

**Figure 1:**
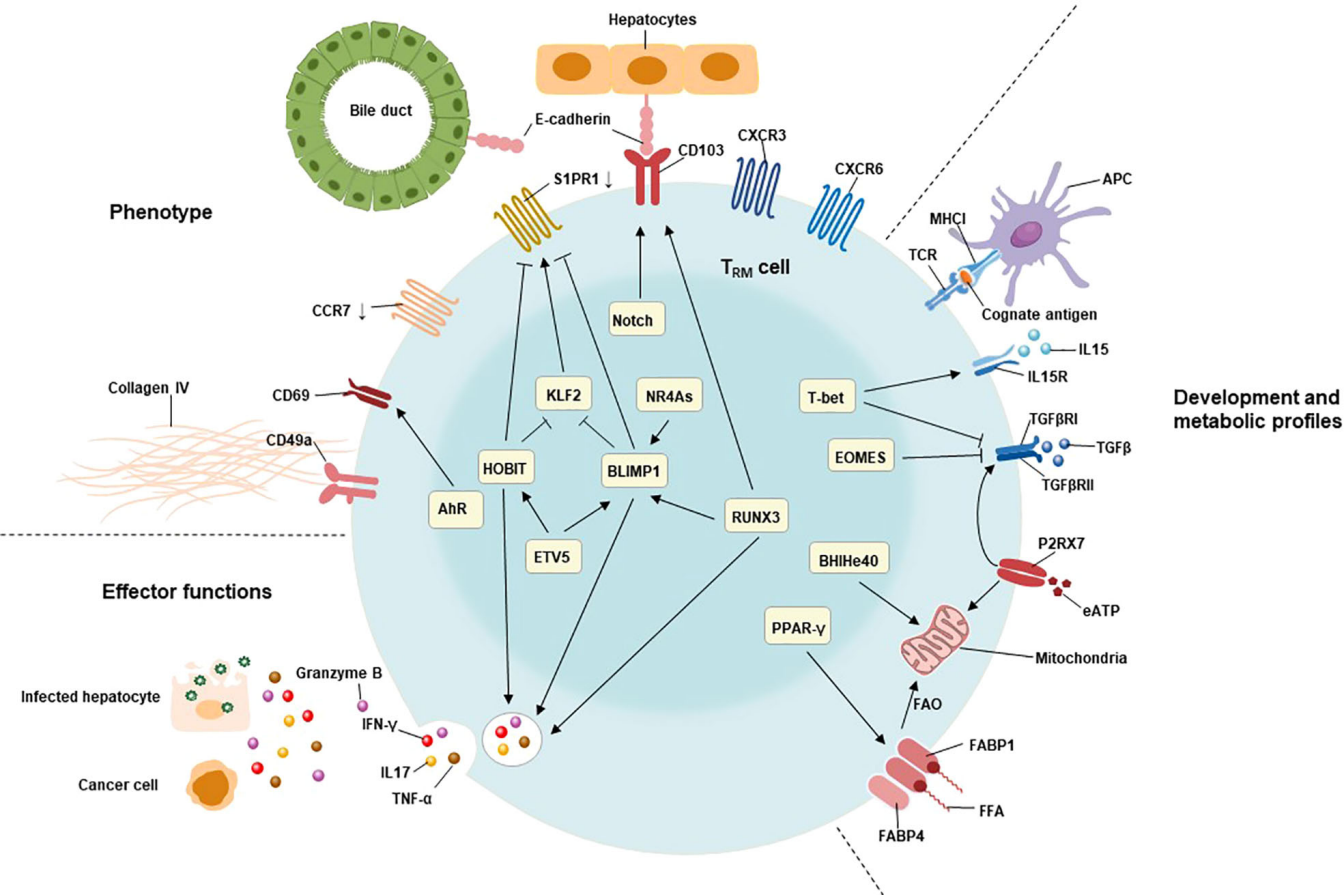
Characteristics of T_RM_ cells include their tissue residency, long-term persistence, and effector function. The residency status of liver T_RM_ cells is regulated by the combined action of B lymphocyte-induced maturation protein 1 (BLIMP1), BLIMP1 homolog in T cells (HOBIT), Notch, and runt-related transcription factor 3 (RUNX3). BLIMP1 and HOBIT downregulate CCR7, Krüppel-like factor 2 (KLF2) and tissue export pathway sphingosine 1-phosphate receptor 1 (S1PR1), while Notch directly upregulates the expression of CD103 on T_RM_ cells. The interaction of CD103 and E-cadherin expressing on hepatocytes as well as cholangiocytes may be involved in adhesion and retention of hepatic T_RM_ cells. Furthermore, the expression of BLIMP1 is regulated by the transcription factor runt-related transcription factor 3 (RUNX3) and NR4A family of orphan nuclear receptors (NR4As). The effector functions of liver T_RM_ cells include direct killing of infected or malignant cells by secreting cytotoxic molecules and inflammatory cytokines, such as granzyme B, TNF-α, IFN-γ, and IL17. The expression of these cytotoxic components is regulated by HOBIT, BLIMP1 and RunX3. The development and maintenance of T_RM_ cells require stimulation with IL15, and TGFβ, as well as cognate antigens presenting by antigen-presenting cells (APC). T-bet is essential for the sustain expression of IL15 receptor, albeit at low levels. Meanwhile, the expression of TGFβ receptor is also regulated by P2X purinreceptor 7 (P2RX7), a sensor for extracellular nucleotides that promotes mitochondrial homeostasis. Mitochondrial fatty acid β-oxidation (FAO) is an important energy source for T_RM_ cells. Peroxisome proliferator-activated receptor-γ (PPARγ) drives the upregulation of FABP1 and FABP4 to promote free fatty acid uptake from the extracellular compartment, while the transcription factor BHlHe40 maintains mitochondrial fitness.

The network of transcription factors underlies the unique features of T_RM_ cells, including liver T_RM_ cells (**Figure 1**). These transcription factors include B lymphocyte-induced maturation protein 1 (BLIMP1; also known as PRDM1), homologue of BLIMP1 in T cells (HOBIT; also known as ZFP683), runt-related transcription factor 3 (RUNX3), Notch, Peroxisome proliferator-activated receptor-γ (PPAR-γ), BHlHe40, TBX21(T-bet), aryl hydrocarbon receptor (AhR), Eomesodermin (EOMES), and NR4A family of orphan nuclear receptors (NR4As). The combined action of these transcription factors contributes to the residency status of liver T_RM_ cells (64, 69).

HOBIT is specifically up-regulated in T_RM_ cells and, together with related Blimp1, mediates the development of T_RM_ cells in lymphoid organs and non-lymphoid tissues (70). The co-expression of HOBIT and BLIMP1 instructs the downregulation of CCR7, transcription factor 7 (TCF7), KLF2, and S1PR1 in T_RM_ cells (71). CCR7 is the receptor for chemokine ligand 19 (CCL19) and chemokine ligand 21 (CCL21) that responsible for cell migration to secondary lymphoid tissues (72).Meanwhile, TCF7, KLF2 and S1PR1 are involved in the tissue egression of lymphocytes (71). Interestingly, KLF2 regulates the expression of S1PR1 in lymphocytes of tissues, which directs them returning to circulation (44). Consequently, the Hobit-Blimp1 transcriptional module retains T_RM_ cells within tissues through silencing the genes related to recirculation in addition to suppressing the markers related to egression. Furthermore, a murine study demonstrated that the transcriptional repressor Capicua (CIC) controls the development of liver T_RM_ cells. Mechanistically, they found that CIC could regulate the expression of HOBIT by inhibiting the ETS variant transcription factor 5 (ETV5) (73). RUNX3 and Notch are essential for the maintenance of T_RM_ cells by repressing the expression of genes involved in the formation of circulating memory T cells and inducing the expression of retention molecules, including CD103 (74). The collaboration of HOBIT, BLIMP1 and RUNX3 also drives immediate effector function in T_RM_ cells by inducing and sustaining granzyme B production (75–77). Notch, predominantly expressed in newly developed T_RM_ cells, not only regulates expression of IFN-γ upon restimulation but also contributes to the mitochondrial fatty acid β-oxidation (FAO) in T_RM_ cells (74, 75). Importantly, exogenous free fatty acids uptake and their FAO are required for the survival and effector function of T_RM_ cells (78). Meanwhile, PPAR-γ facilitate the uptake of free fatty acids by upregulating fatty acid binding proteins 1 and 4 (FABP1 and FABP4) in T_RM_ cells (78, 79). Bhlhe40, a stress-responsive protein, promotes the survival and function of T_RM_ cells under stress conditions by sustaining mitochondrial fitness (80).

T-bet is crucial to sustain the expression of the IL15 receptor β subunit (IL15Rβ) and therefore enable the long-term lineage stability of T_RM_ cells, albeit at low levels (81). Activation of aryl hydrocarbon receptor (AhR) may be associated to the maintenance of liver T_RM_ cells by increasing the expression of CD69 (82, 83).Recent studies reveal that EOMES directly inhibited expression of IFN-γ *in vitro*, while EOMES deletion in T cells led to substantially increased frequency and percentage of T_RM_ precursor in the liver (84, 85). Therefore, the downregulation of EOMES in T_RM_ cells is required to not only their formation, but also their effector function.

Additionally, the NR4As are composed of NR4A1 (Nur77), NR4A2 (Nurr1), and NR4A3 (Nor1). During the memory phase of influenza infection, Nur77 deficiency in CD8^+^ T cells reduces the frequency of CD8^+^ T_RM_ cells in the liver without any effect on lung or bone marrow CD8^+^ T_RM_ cells and other memory CD8^+^ T cells such as T_CM_ and T_EM_ (86), indicating a specific role of Nur77 on liver T_RM_ cell differentiation. In addition, the expression of the transcription factors involving in T_RM_ differentiation (BLIMP1 and T-bet) is decreased, while the expression of EOMES is increased in absence of Nor1 in CD8^+^ T cells (87). Interestingly, NR4As are particularly enriched in the highly functional CD28^+^ subset of CD8^+^ T_RM_ cells. Importantly, deficiency of Nurr1 specifically reduces the percentage of these CD28^+^ T_RM_ subsets (88). To conclude, NR4As are important regulators involved in the differentiation of CD8^+^ T_RM_ cells. However, not all NR4As are comprehensively interrogated at the specific differentiation steps of CD8^+^T_RM_ cells. Therefore, figure out which signals promote the expression of NR4As in addition the role of NR4As in CD8^+^ T_RM_ cell differentiation await further investigation.

Although these transcription factors described above have been shown to be critical for T_RM_ cells, it is difficult to determine which are the specific key regulators of T_RM_ differentiation and maintenance, as they are also expressed in other CD8^+^ effector or memory subsets. Therefore, the differentiation and maintenance of T_RM_ may be regulated by the cooperation of multiple transcription factors.

### Effector functions

Similar to other tissue T_RM_ cells, liver T_RM_ cells also have timely, potent and durable effector functions. When pathogens enter the liver, T_RM_ cells can take advantage of tissue residency to generate a rapid and effective protective immune response by secreting multiple chemokines and cytokines in a deployment-ready mode (75). The cytotoxic cytokines enable them to directly eliminate infected or malignant cells as well as control invading pathogens, while chemokines and pro-inflammatory cytokines recruit and activate other immune cells, thereby remodeling the local liver microenvironment for more potent effector functions. Furthermore, liver CD8^+^ T_RM_ cells express high levels of Ki-67 and TCF1, showing their proliferative and self-renewal potential (89). Actually, T_RM_ cells can persist in the liver for years and exert durable protective effect (17). In addition, T_RM_ cells may help to significantly promote the repopulation of locally resident and circulating memory T cells after infection, suggesting their role in establishing secondary memory T cells to prevent future reinfection with the same pathogen (90, 91). Accordingly, T_RM_ cells have been used to develop vaccines that generate stronger and longer-lasting immune responses than conventional vaccines (15, 28, 29). Meanwhile, CD8^+^ T_RM_ cells are able to attract hepatic stellate cells (HSCs) in a CCR5-dependent manner and predispose activated HSCs to FasL-Fas-mediated apoptosis, thereby promoting liver fibrosis regression (39). However, every coin has two sides, as do liver T_RM_ cells. Once T_RM_ cells are interfered by cognate antigens and damage hepatocytes and cholangiocytes, it may lead to the occurrence of AILD. Meanwhile, auto-aggressive liver CXCR6^+^CD8^+^ T_RM_ cells cause hepatic immune pathology in NASH in an MHC-class-I-independent manner (47). Therefore, clarifying the biological characteristics and development of liver T_RM_ cells so as to accurately manipulate liver T_RM_ cells can enhance the effector functions of T_RM_ cells and avoid weaknesses.

## Development of liver T_RM_ cells

Multiple factors including T cell-intrinsic and environmental factors are believed to be involved in the T_RM_ cell differentiation. Thereinto, the first question to be addressed is the origin of T_RM_ cells. Olivier, O et al. analyzed antigen-activated T cells from different tissues using TCR sequences. They found that T_CM_ cells in the lymph nodes share a common clonal origin with T_RM_ cells (92), indicating that these subsets derive from the same naïve T cell precursors. Moreover, the differ in TCR stimulation affinity, namely the strength of antigen binding of TCRs, affects the subsequent development of T_RM_ cells (93). In this regard, high TCR affinity leads to T_EM_ development, whereas a low TCR affinity results in short-lived memory cells with impaired secondary immune response (94, 95).It is reported that T_RM_ cells have different TCR stimulation affinity compared to splenic memory T cells (93, 94). Furthermore, there is heterogeneity in the magnitude of TCR stimulation affinity required for the development of functional CD8^+^ T_RM_ cells in different tissues (93, 96). For example, Maru, S et al. demonstrated that brain T_RM_ cells stimulated with suboptimal stimulation strength respond more effectively to CNS infection than cognate antigen, suggesting that the strength of antigen stimulation affects the functional integrity of TRM cells in a persistent viral infection (93). However, the specific strength of TCR stimulation affinity required for inducing liver-adapted T_RM_ cells has not been determined.

Additionally, the killer cell lectin-like receptor G1 (KLRG1) may contributes to figure out the source of T_RM_ cells. KLRG1 is upregulated in short-lived effector cells (SLECs, KLRG1^hi^ IL7Rα^lo^), whereas the memory precursor effector cells (MPECs) that turn into heterogenous populations of memory CD8^+^ T cells, bear negative or low expression of KLRG1 (9, 97). Adoptive transfer experiments have shown that MPECs could generate T_RM_ cells after entering specific tissues (98). In addition, a portion of KLRG1^+^ CD8^+^ T cells can downregulate KLRG1 during the contraction phase of immune response and differentiated into T_RM_ cells. The latter subset accounts for approximately half of the liver T_RM_ cell population and has a stronger cytotoxic and proliferative capacity than those directly derived from KLRG1^-^CD8^+^ T cells (99). These findings suggest that liver T_RM_ cell can originate from both KLRG1^+^ or KLRG1^-^ lymphocytes.

On the other hand, studies have shown that cognate antigens and inflammatory cytokines also contribute to the development and maintenance of liver T_RM_ cells (**Figure 1**).

Antigenic challenge induces and amplifies antigen-specific T_RM_ cell proliferation, and maintained at low-level magnitude in the liver T_RM_ pool after the clearance of infection. Actually, the capacity of hepatic T_RM_ niches is large enough to lodge multiple T_RM_ cells with different specificities without displacing previously established cells (14). Therefore, newly formed liver T_RM_ cells do not displace existing T_RM_ cell populations (14). Intriguingly, T_RM_ cells induced by cognate antigen in secondary immune response are mainly developed from the pre-existing T_RM_ populations, instead of circulating memory T cells (90, 100). Therefore, cognate antigens contribute to the immune response mediated by T_RM_ cells and the construct of polyclonal T_RM_ cell repertoire.

The differentiation and development of liver T_RM_ cells can be mediated by multiple cytokines, including IL2, IL15, TGFβ and IL10. IL2 is mainly produced by activated T cells. It promotes the growth, proliferation and differentiation of lymphocytes, and is essential for the body’s immune response and antiviral infection. Interestingly, human liver CD8^+^ T_RM_ cells express high levels of IL2 (17, 36). The unusually high IL2 production of hepatic CD8^+^ T_RM_ may be important for their protective potential, as autocrine IL2 is needed to the persistence of memory responses to pathogens and secondary population expansion of CD8^+^ memory T cells (17, 101). In addition, IL15 is known to be involved in T_RM_ development and longevity. Although shares a receptor subunit with IL2, IL15 has a perceptible difference in immunomodulatory properties. Generally, IL15 induces the proliferation and survival of circulating memory CD8^+^ T cells (102, 103). Nevertheless, the upregulation of the IL15 receptors in memory CD8^+^ T cells indicating that IL15 stimulation may be essential for T_RM_ development (102). It was reported that IL15 was able to induce CD69, CXCR3 and CXCR6 expression on peripheral CD8 T cells in a dose-dependent manner, all of which were highly expressed on hepatic T_RM_ cells (17). Consistently, IL15 knockout mice prevent CD8^+^ T_RM_ cells development in the liver (14). Meanwhile, the expression of hepatic IL15 is positively correlated with T_RM_ cells in AIH liver (36). Therefore, the presence of IL15 may be essential for the formation of liver T_RM_ cells. Another important cytokine is the TGFβ. TGFβ is a pleiotropic cytokine that is produced in an inactive form, namely latency associated peptide (LAP). LAP can be activated by binding to integrin αvβ6 on epithelial cells and/or integrin αvβ8 on dendritic cells and endothelial cells (104). Activated-TGFβ induces CD8^+^ T_RM_ cells to express CD103 as well as downregulate of EOMES (81, 98), which are mandatory for their generation, adhesion and long-term persistence in the liver. In fact, TGFβ is capable of inducing liver-adapted T_RM_ cells, and importantly, hepatic TGFβ is significantly correlated with T_RM_ cells infiltration in human liver (17, 18, 36). Actually, sequential exposure to IL-15 followed by TGFβ efficiently induced *de novo* CD69^+^CD103^+^CD8^+^ T_RM_ cells, with similar frequencies to those found in healthy livers (17). These studies suggest that the expression of IL15 and TGFβ in the liver promotes the development and residency of CD103^+^ T_RM_ cells in human. However, a recent mouse experiment showed that constitutive TGFβ signaling did not accelerate the development of liver T_RM_ cells (105), indicating that TGFβ may have functional heterogeneity in liver T_RM_ cells between human and mice. Meanwhile, monocyte-produced IL10 induced the release of surface-bound TGFβ of antigen-presenting cells, while blocking IL10 reduced CD103 expression on T_RM_ cells (106). Therefore, IL10-mediated TGFβ signaling may have a critical role in the generation and retention of liver T_RM_ cells.

Additionally, several cytokines have been reported to be involved in T_RM_ development outside the liver. For example, the IFN-β and IL12 are described to positively influence T_RM_ cells differentiation by regulating the expression of CD103 and CD69 in the intestine (107). Meanwhile, it is reported that hair follicle-derived IL7 is involved in CD4^+^ T_RM_ cells generation and persistence in the skin (108, 109). Intriguingly, hepatocytes are the main source of IL7 in the liver, and the hepatocyte-derived IL7 can promote the survival of memory CD4^+^ and CD8^+^ T cells (110). However, the specific role of these cytokines on the development of liver T_RM_ cells remains to be elucidated.

## Metabolic profiles of liver T_RM_ cells

There are significant differences in the metabolic profiles of different T cell subsets. Several studied demonstrated that preferences for certain metabolic pathways for energy affect T_RM_ cells generation, tissue retention, and effector functions.

Generally, highly proliferative and active cells prefer the glycolytic pathway, while quiescent cells primarily use oxidative phosphorylation and FAO to generate ATP. Thereinto, mammalian target of rapamycin (mTOR), including two subunits of mTOR complex 1 (mTORC1) and mTORC2, is a key regulator involved in regulating T cell nutrient metabolism, proliferation and activation (111). While activating, it induces glucose consumption to support T cell proliferation. There is strong evidence that mTOR plays an important role in the generation of T_RM_ cells (112). Rapamycin, an mTORC1 inhibitor, has been reported to induce the formation of memory CD8^+^ T cells but reduce T_RM_ production in the gut, thereby protecting mice from functional CD8^+^ T_RM_ cell-mediated intestinal autoimmunity (113). However, the exact effects of rapamycin on the liver T_RM_ cells are still under investigation.

Fatty acid binding proteins (FABPs) are a group of intracellular molecules that mediate the trafficking and metabolism of fatty acids (114). Reliance on FAO has recently been shown to be essential for the development and maturation of CD8^+^ T_RM_ cells (78). For example, studies on skin T_RM_ cells revealed that T_RM_ cells upregulate FABP4 and FABP5 so as to uptake and utilize exogenous free fatty acid (FFA) as an energy source for their survival. Consistently, the deficiency of FABP4 and FABP5 results in impaired functional properties and longevity of skin CD8^+^ T_RM_ cells, but not influence the survival of T_CM_ cells *in vivo (* 78). However, T_RM_ cells from different tissues express distinct FABPs with selected in a tissue-specific fashion that is optimized for local fatty acid availability (78, 79). It has been demonstrated that liver T_RM_ cells express high levels of FABP1 and a low concentration of FABP4, but do not express FABP5 (79). In a murine model of LCMV infection, FABP1 deficiency mice manifested impaired T_RM_ cell development in the liver but not in the skin. Furthermore, the selective loss of liver T_RM_ cells could be restored upon re-expression of FABP1 (79). Interestingly, bezafibrate, the PPAR agonists that promote FAO, has been confirmed to improve the effector function of memory T cells (115). Therefore, a unique FAO regulator, FABP1, driven by a liver-specific microenvironment may be a promising target for intervention in hepatic T_RM_ cells.

Additionally, several studies revealed that P2X purinreceptor 7 (P2RX7) is required for the establishment, maintenance and functionality of T_RM_ cells. P2RX7 is a sensor for extracellular nucleotides that promotes mitochondrial homeostasis and metabolic function of memory CD8^+^ T cells (116). Importantly, P2RX7 supports T_RM_ development by enhancing CD8^+^ T cell sensing of TGFβ *via* upregulate the TGFβ receptor II (TGFβRII) through calcineurin signaling. Meanwhile, P2RX7-deficient T_RM_ cells progressively decayed and expressed dysregulated T_RM_-specific markers such as CD103. Consistently, upregulation of TGFβRII expression rescued P2RX7-deficient T_RM_ cell generation as well as mitochondrial function (116), indicating that sustained P2RX7 signaling is required for long-term T_RM_ cell maintenance. However, another study demonstrated that P2RX7 activation in sterile tissue damage during acetaminophen-induced liver injury selectively enhanced the NAD-induced cell death of liver T_RM_ cells compared with circulating T cells, whereas concurrent TCR engagement promoted survival of T_RM_ cells (117).These studies suggest that differences in genetic background, microbiota as well as their metabolites might have caused discrepancies in the regulation of T_RM_ differentiation and maintenance by P2RX7.

## Liver T_RM_ cells in the chronic liver disease

The porous epithelial layer is a unique feature of the liver, which not only enables the direct interaction of T_RM_ cells with hepatocytes, but also facilitates the encounter of cognate antigens by T_RM_ cells in the liver. T_RM_ cells that reside in the unique microenvironments of the liver not only develop in response to infection, such as viral or parasite infection, but are also detected in AILD, NAFLD, HCC and liver allografts. Below, we discuss the unique characteristics of T_RM_ cells in the local microenvironment of different chronic liver diseases, their role in disease progression, as well as their potential therapeutic value (**Table 1**).

### Liver T_RM_ cells in viral infection

Hepatoviral infection is mainly caused by the hepatitis B (HBV) and hepatitis C (HCV) viruses and the course can be acute or chronic. Chronic infection with hepatotropic virus can cause liver damage, cirrhosis, liver failure, development of HCC, and even liver transplantation. It has been demonstrated that hepatic T_RM_ cells play a major antiviral immune response during chronic hepatic virus infections.

Pallett, J et al. were the first to report the virus-specific liver CD8^+^ T cells in chronic HBV infection, in which approximately 90% of them have a T_RM_ cell-like phenotype (CD69^+^CD103^+^ or CD69^+^CD103^−^) (17). CD8^+^ T_RM_ cells can persist in the liver for several years after primary infection and expand in patients with HBV. Importantly, virus-specific CD8^+^ T_RM_ cells could still be detected in spontaneously recovered HBV patients, with effector functions equivalent to those from chronic HBV-infected patients (18), suggesting the long-term viral control of hepatic CD8^+^ T_RM_ cells. Virus-specific CD8^+^ T_RM_ are very efficient in their function. During HBV viral infection, PD-L1 expression is upregulated in hepatic sinusoidal endothelial cells and hepatocytes (118). PD-L1 on intrahepatic cells can interact with PD1 on T_RM_ cells, thereby dampening pro-inflammatory T_RM_ cell responses (19). Nevertheless, even though T_RM_ cells express high levels of the PD1, they readily produce IFN-γ, TNF-α, perforin, and IL2 upon stimulation (17). IFN-γ and TNF-α mediated control of HBV replication, while perforin may contribute to the directly elimination of infected hepatocytes (20, 21). Furthermore, IL2 production is most strikingly enhanced within CD69^+^CD103^+^ T_RM_ cells, which contributes to overcome PD-L1-mediated inhibition and exhaustion, stressing their ability for survival and maintenance (21, 119). Additionally, CD8^+^ T_RM_ cells are enriched in HBV patients who achieved viral control, and their abundance is inversely correlated with HBV viral load, stressing that the virus-specific liver T_RM_ cells can control viral replication and contribute to the functional cure for HBV patients (17, 22). Therefore, liver T_RM_ cell expansion may be a potential therapeutic target for chronic HBV infection.

Additionally, a portion of HBV patients are co-infected with hepatitis D virus (HDV), which often indicates a poor prognosis. As the smallest known human virus, HDV has perfectly adapted to escape recognition by CD8^+^ T cells restricted by common human leukocyte antigen (HLA) class I alleles (120). A recent study suggested that antigen-nonspecific activation of hepatic CD8^+^ T_RM_ cells may be involved in intrahepatic inflammation and disease progression in HDV infection (121).

CD8^+^ T_RM_ cells also play an essential role in long-term antiviral response in chronic HCV infection (23–25). In the chimpanzee model of HCV reinfection, depletion of CD8^+^ T cells resulted in prolonged the virus persistence and prevented effective viral clearance, while recovery of CD8^+^ T cells lead to virus eradication (26). Meanwhile, a large number of CD69^+^CD8^+^ T cells were detected in the liver of animals recovered after HCV infection, but not in the peripheral blood. These subsets may be hepatic T_RM_ cells, which are required for protection from persistent HCV Infection (26). Consistently, liver CD8^+^ T_RM_ cells are highly increased in chronic HCV patients and possess a specific activation and cytolytic potential and are important in controlling chronic HCV infection (27).

Besides hepatotropic virus infection, liver CD8^+^ T_RM_ cells contribute to the effective clearance of Lymphocytic choriomeningitis virus (LCMV) as well. In the murine model of LCMV infection, virus-specific T_RM_ cells in the liver could be influenced by other liver-resident immune cells. For example, deficiency of liver-resident natural killer (LrNK) cells increased both the frequency and antiviral activity of hepatic T_RM_ cells *via* the interaction of PD1 and PD-L1. Consistently, transfer of LrNK cells into LrNK-cell-deficient mice as well as PD-L1 inhibition restrain hepatic T_RM_ cell function, resulting in impaired viral clearance (122). Furthermore, during LCMV infection, other liver-resident T cells, such as γδ T cells, also expand and promote viral clearance by producing IFN-γ and TNF-α (123).

Current studies suggest that hepatic T_RM_ cells may be involved in the clearance of viral infection, protect patients from persistent viral infection, and improve disease prognosis. However, the role of TRM cells in different viral infections in the liver remains to be further elucidated.

### Liver T_RM_ cells in parasite infection

Besides viral infections, several studies have investigated the role of liver T_RM_ cells in parasitic infections, including malaria and leishmaniasis.

Malaria is an insect-borne infectious disease caused by the infection of *Plasmodium* through the bite of *Anopheles* mosquitoes or the transfusion of the blood of a person carrying Plasmodium (124). *Plasmodium* has a complex life cycle, including three stages in the liver, blood and mosquito. During infection of malaria, *Plasmodium* promotes the development of antigens-specific T_RM_ cells (16, 125–127). These T_RM_ cells could mediate protective immune responses through killing infected cells by producing pro-inflammatory cytokines, such as IFN-γ and TNF-α (16, 30). Additionally, T_RM_ cell depletion abrogated an efficient immune response to a murine model of *Plasmodium* infection (31).Due to the protective immune response of T_RM_ cells against malaria, vaccination strategies that maximize intrahepatic *Plasmodium*-specific T_RM_ development have emerged (16, 28, 29, 32–34, 127). An example is the *Plasmodium* ribosomal protein vaccine (15). One of the antigens for this vaccine is PbRPL6_120-127_, a highly conserved H2-K^b^-restricted epitope from the 60S ribosomal protein L6, expressed throughout the parasite life cycle, across *Plasmodium* species (15). It may be an optimal antigen for endogenous liver T_RM_ development and protection against malaria. A single dose of this vaccine could provide effective and prolonged sterilizing immunity against high dose sporozoite challenges (15). Indeed, people living in malaria-endemic areas do not acquire effective protection against reinfection from malaria (128), while attenuated *Plasmodium falciparum* sporozoite (SPZ) vaccine is highly protective against controlled human malaria infection 3 weeks after immunization (129), suggesting multiple, complex factors are likely responsible for the lack of development of sterilizing immunity to malaria through natural infection. Furthermore, the protection and long-term efficacy of existing vaccines are not satisfactory. Accordingly, to improve the T_RM_-based vaccination against malaria in human, further investigation of the mechanisms that mediate *Plasmodium*-specific T_RM_ generation and function, assessment of the feasibility of currently known antigens, as well as identification of novel target epitopes are required.

Recently, the role of T_RM_ cells in Leishmaniasis was studied as well. Leishmaniasis is a zoonotic disease caused by Leishmania, which can cause cutaneous and visceral kala-azar in humans (130). There are various types of Leishmania in which Leishmania infantum (L. infantum) primarily infects the liver (131–133). During chronic L. infantum infection, liver T_RM_ cells are generated and play a protective role. Importantly, induction by the Leishmania proteins LirCyP1 and LirSOD promotes the expansion of hepatic T_RM_ cells, which could be a promising strategy for prophylactic or therapeutic vaccine formulations (131).

Taken together, hepatic T_RM_ cells are critical in parasitic infections, and the T_RM_-based vaccination strategies could hold remarkable promise in providing long-term protection.

### Liver T_RM_ cells in AILD

AILD is a group of liver inflammatory damage diseases mediated by abnormal autoimmunity, including autoimmune hepatitis (AIH), primary biliary cholangitis (PBC), primary sclerosing cholangitis (PSC), IgG4-related sclerosing cholangitis (IgG4-SC), etc. AIH is an inflammatory liver disease dominated by T cell-mediated hepatocyte injury. Antigen-specific CD8^+^ T_RM_ cells have been reported to characterize the liver tissue of subjects with indeterminate pediatric acute liver failure (PALF) and may serve as a novel biomarker for PALF due to AIH (37, 38). Recently, our group demonstrated that CD69^+^CD103^+^CD8^+^ T_RM_ cells play an important role in the pathogenesis of AIH, and histological remission is accompanied by decreased hepatic CD8^+^ T_RM_ cells in AIH patients (36). In addition, hepatic CD8^+^ T_RM_ cells from AIH patients expressed a higher level of PD-1, CXCR3 and granzyme B than those of healthy controls. Consistently, in AIH liver, both expression of IL15 and TGFβ, cytokines that induce T_RM_ cells *in vitro*, were elevated, suggesting that the immunological microenvironment facilitates hepatic CD8^+^ T_RM_ cells development and residency (36). Intriguingly, E-cadherin, the natural ligand of CD103, is widely expressed in hepatocytes of AIH patients, and located closely to CD8^+^ T_RM_ cells, which may contribute to the residency of CD8^+^ T_RM_ cells in the liver. Furthermore, E-cadherin is also widely expressed in cholangiocytes (53, 54), suggesting that CD103^+^ T_RM_ cells may be involved in pathology of bile duct injury in cholestatic liver diseases, such as PBC and PSC. Interestingly, a recent study on biliary immune atlas revealed the presence of CD8^+^ T_RM_ cells in areas of biliary inflammation in PSC patients (134).

### Liver T_RM_ cells in NAFLD

Nonalcoholic fatty liver disease (NAFLD) is considered a hepatic manifestation of metabolic syndrome, hypertension and type 2 diabetes. Several studies have demonstrated that liver-resident T cells and the proinflammatory immune response they elicit are involved in NAFLD disease progression (135–138). Generally, liver-resident γδT cells induce chronic liver inflammation by producing proinflammatory cytokines such as IL17A, IFN-γ, and TNF-α, contributing to the pathogenic immune response to NAFLD (123, 137, 139). Furthermore, systemic inflammation in obese patients is associated with increased T_RM_ cells in the liver and may be further involved in NAFLD disease progression. Importantly, activated T_RM_ cells are significantly increased in the liver and visceral fat of obese patients. These activated T_RM_ cells produce multiple pro-inflammatory cytokines, such as IL1β, IL2, IL12, and IL15 (140), further contributing to the generation of T_RM_ cells in addition to the overall pro-inflammatory phenotype in obese patients.

Interestingly, a recent study revealed that CD69^+^CD103^-^CD8^+^ T_RM_ cell may perform a protective role in resolving liver fibrosis of nonalcoholic steatohepatitis (NASH) (39). They demonstrated that the reduction of these CD8^+^ T_RM_ cells significantly delayed fibrosis resolution *via* influencing predisposed HSCs apoptosis, while adoptive transfer of these cells protected mice from fibrosis progression in a CCR5-dependent manner (39). Therefore, the paradoxical roles of T_RM_ cells in NAFLD and their specific mechanisms remain to be further investigated.

### Liver T_RM_ cells in HCC

HCC accounts for the majority of primary liver cancers and is currently one of the leading causes of cancer-related deaths worldwide. The development of HCC is a complex multistep process caused by multiple risk factors, whereas the function of tumor-infiltrating T cells is important for moderating antitumor immunity in HCC development and determining the clinical fate of HCC patients (40). There are strong evidences that CD103^+^ T_RM_ cells are enriched in HCC patients and associated with better prognosis (19, 41, 42).

In murine model of HCC, hepatic T_RM_ cells were significantly expanded, and their frequencies decreased during HCC progression (141). Meanwhile, hepatic T_RM_ cells in HCC have an exhausted phenotype, manifested by expression of PD1, LAG3, and TIM3 (40). Given that PD1 expression in T_RM_ cells in HCC is associated with poor disease outcome (142), immunotherapy targeting checkpoint inhibition has been applied to HCC (143, 144). During immunotherapy for HCC, PD1^high^ T_RM_ cells are the most sensitive cells to anti-PD-1 therapy to overcome tumor growth and progression (145). Additionally, other markers of exhaustion and inhibition, such as TIM3 and CTLA4, and pro-inflammatory cytokines, such as IFN-γ and TNF-α, can also be simultaneously expressed on T_RM_ cells in HCC patients (142), suggesting that hepatic T_RM_ cells may be involved in direct killing of tumor cells. Overall, hepatic T_RM_ cells might play an extremely important role in both HCC development and anti-tumor therapy.

### Liver T_RM_ cells in transplantation

Liver transplantation is the treatment of last option for end-stage liver disease of various causes and severe acute liver failure. It has been reported that donor-derived T_RM_ cells are detectable in the liver allografts and that their abundancy could be correlated with organ survival and reduced rejection (146–148). Specifically, long-term persistence of lung donor-derived T_RM_ cell is associated with reduced incidence of clinical events that precipitate allograft injury, including primary graft dysfunction (PGD) and acute cellular rejection (ACR) (149). However, the association of liver donor-derived T_RM_ cells with the incidence of clinical events remains to be further elucidated (150). In liver allograft tissues, approximately 2-6% of CD8^+^ T cells had a donor-derived T_RM_ phenotype at 11 years post-transplantation (18), well demonstrating the longevity of human liver T_RM_ cells. Additionally, donor-derived T_RM_ cells from an HBV-infected liver allograft could migrate to draining lymph nodes with down-regulation of some T_RM_-specific markers. However, they were not detectable in blood vessels (18). Interestingly, the same study demonstrated that a lower quantity of recipient-derived virus-specific T cells with a T_RM_-like phenotype were detected in the liver and blood (18), further revealing the extrahepatic origin of T_RM_ cells in the liver. Nevertheless, CMV-specific T_RM_ cells in human liver allografts did not acquire a T_RM_ phenotype in the liver, possibly due to the lack of relevant antigens in the liver.

## Perspectives

The tissue retention and longevity of hepatic T_RM_ cells and their potent effector functions demonstrate their potential role in chronic liver diseases. The above studies have shown that hepatic T_RM_ cells play a protective role in viral and parasitic infection, NAFLD, HCC, and liver transplantation, whereas they might be pathogenic in AILD such as AIH. However, further studies are needed to reveal more mechanisms of T_RM_ cell biology, including the phenotype of T_RM_ cells and the specific mechanisms that regulate their development and differentiation. Furthermore, there are several key points regarding hepatic T_RM_ cells that remain to be investigated.

Firstly, T_RM_ cells are heterogeneous, and the subsets of T_RM_ cells that function in the liver under different conditions will differ in the expression of surface markers and biological behavior. For example, the predominant T_RM_ cells associated with the pathogenesis of AIH are CD8^+^CD69^+^CD103^+^ T_RM_ cells that highly express PD1, CXCR3 and granzyme B (36); whereas liver T_RM_ cells of patients with acute hepatitis A are mainly CD8^+^CD69^+^CD103^-^ T_RM_ cells that express high levels of HIF-2α (55). T_RM_ cells are essential for the adaptive immune response. While interfering different chronic liver diseases by hepatic T_RM_ cells, the biological function and disease specificity of the corresponding T_RM_ cells should be carefully considered. Therefore, identifying the specific subsets of hepatic T_RM_ cells that play a major role in the chronic liver diseases will help to define precise future intervention strategies.

Secondly, since the liver is an immune organ, we should pay attention to the crosstalk of other immune cells in the liver to hepatic T_RM_ cells. Clarify whether they are cooperative or antagonistic is of great significance. It has been shown that LrNK cells can reduce the frequency and antiviral activity of hepatic T_RM_ cells through the interaction of PD1 and PD-L1 during LCMV infection (122). However, the interaction among other liver-resident cells remains to be further investigated. For example, liver-resident γδ T cells, participate in the pathogenic immune response to NAFLD by producing proinflammatory cytokines (123), are capable of form a long-lived resident memory-like subpopulation upon local inflammation or infection. Nevertheless, it is still unclear whether there is crosstalk between unconventional γδ T_RM_ cells and conventional αβ T_RM_ cells. Accordingly, clarifying these interactions will shed light on the overall immune homeostasis of the liver and lay the groundwork for developing holistic therapies.

Thirdly, given that the biliary system that communicates with the digestive tract and the portal blood that flows directly into the liver may contain various gut-derived microorganisms as well as their metabolites, hepatic T_RM_ cells are chronically exposed to, and may be trained by them. Whether the composition of the gut microbiome, specific species of the gut microbiome or their metabolites would influence the phenotype and development of hepatic T_RM_ cells are unknown yet. Elucidating these interactions may open up new avenues for the realization of therapeutic strategies for “enteric treatment of liver disease”.

To conclude, hepatic T_RM_ cells are considered to play a crucial role in various chronic liver diseases. Elucidating and characterizing the underlying mechanisms of hepatic T_RM_ cells will shed light on the control of chronic liver diseases and provide promising strategies for precision immunotherapy in different chronic liver diseases.

## Author contributions

All authors listed have made a substantial, direct, and intellectual contribution to the work and approved it for publication.

## Conflict of interest

The authors declare that the research was conducted in the absence of any commercial or financial relationships that could be construed as a potential conflict of interest.

## Publisher’s note

All claims expressed in this article are solely those of the authors and do not necessarily represent those of their affiliated organizations, or those of the publisher, the editors and the reviewers. Any product that may be evaluated in this article, or claim that may be made by its manufacturer, is not guaranteed or endorsed by the publisher.
